# Drug-Induced Hematologic Syndromes

**DOI:** 10.1155/2009/495863

**Published:** 2009-07-07

**Authors:** David M. Mintzer, Shira N. Billet, Lauren Chmielewski

**Affiliations:** Section of Hematology and Medical Oncology, Pennsylvania Hospital, Philadelphia, PA 19106, USA

## Abstract

*Objective*. Drugs can induce almost the entire spectrum of hematologic disorders, affecting white cells, red cells, platelets, and the coagulation system. This paper aims to emphasize the broad range of drug-induced hematological syndromes and to highlight some of the newer drugs and syndromes. *Methods*. Medline literature on drug-induced hematologic syndromes was reviewed. Most reports and reviews focus on individual drugs or cytopenias. *Results*. Drug-induced syndromes include hemolytic anemias, methemoglobinemia, red cell aplasia, sideroblastic anemia, megaloblastic anemia, polycythemia, aplastic anemia, leukocytosis, neutropenia, eosinophilia, immune thrombocytopenia, microangiopathic syndromes, hypercoagulability, hypoprothrombinemia, circulating anticoagulants, myelodysplasia, and acute leukemia. Some of the classic drugs known to cause hematologic abnormalities have been replaced by newer drugs, including biologics, accompanied by their own syndromes and unintended side effects. *Conclusions*. Drugs can induce toxicities spanning many hematologic syndromes, mediated by a variety of mechanisms. Physicians need to be alert to the potential for iatrogenic drug-induced hematologic complications.

## 1. Introduction

Hematological disorders arise through a variety of mechanisms and etiologies. Drug-induced hematological disorders can span almost the entire spectrum of hematology, affecting red cells, white cells, platelets, and the coagulation system. Most recent reviews of drug-induced hematological disorders focused on specific drugs or cytopenias. The purpose of this review is to emphasize the broad range of drug-induced hematological syndromes and to highlight some of the newer drugs and syndromes described. However, due to space limitations, this review is not meant to be comprehensive of all drug-induced hematological dyscrasias.

## 2. Immune Hemolytic Anemia

Immune Hemolytic Anemia (IHA) is characterized by destruction of red cells by antibodies acting against antigens on the erythrocyte membrane. Mediated by either IgG or IgM antibodies, IHA may be idiopathic, or secondary to infections, autoimmune diseases, lymphoproliferative disorders, or drugs. Patients present with anemia, reticulocytosis, indirect hyperbilirubinemia, elevated LDH with a positive Coombs test.

Drug-induced IHA may be associated with either drug-dependent or drug-independent antibodies [1]. Other drugs may cause nonimmunologic protein adsorption onto drug-treated red cells. With drug independent autoantibodies, typified by alpha-methyl DOPA, IHA can persist at length, even after the drug is withdrawn. IHA has been described with cephalosporins, nonsteroidal anti-inflammatory agents, levaquin, oxaliplatin, and teicoplanin, amongst others [1, 2].

Intravenous Rh (D) immune globulin, used for treatment of immune thrombocytopenic purpura in non-splenectomized Rh (D)-positive patients, intentionally induces a mild hemolysis, which likely accounts for its mechanism of action. However, severe hemolysis with renal insufficiency, disseminated intravascular coagulation, and death has been reported in a small number of cases [3].

Fludarabine, a purine nucleoside chemotherapeutic agent, has been reported to precipitate or exacerbate the auto-immune hemolytic anemia associated with chronic lymphocytic leukemia. However, combining fludarabine with rituximab and cyclophosphamide may reduce that risk [4].

## 3. Nonimmune Hemolytic Anemias

G6PD deficiency is the most frequent red cell enzymopathy associated with hemolysis. Hemolysis may be precipitated by infection, fava beans, and drugs. The sensitivity to various drugs depends on the inherited mutation and the associated degree of deficiency. In most cases, drug-induced hemolysis is self-limited. The deficiency is X-linked, so manifested more commonly and severely in males. Primaquine, phenazopyridine, nitrofurantoin, and certain sulfas have been associated with hemolysis [5].

Ribavirin, used with peginterferon for treatment of hepatitis C, has been associated with anemia. Ribavirin concentrates within red blood cells, depletes ATP, and promotes hemolysis via oxidative membrane damage. While the anemia will improve by stopping or dose-reducing ribavirin, such strategies may compromise the efficacy of the antiviral therapy. Erythropoietin has been reported to be helpful in moderating the anemia [6].

## 4. Methemoglobinemia

In approximately 3% of the body's hemoglobin, the ferrous iron in heme is oxidized upon deoxygenation, creating methemoglobin. Most of this naturally occurring methemoglobin is reduced to hemoglobin through the methemoglobin reductase enzyme system. Methemoglobinemia, characterized by excess production of methemoglobin, causes impairment in the transport of oxygen. Methemoglobinemia can be congenital (due to defects in enzymatic reduction of hemoglobin) or acquired. Patients present with symptoms of anoxia, cyanosis, reduced oxygen saturation, and chocolate-brown arterial blood. Confirmation of the diagnosis is made by measurement of methemoglobin on arterial blood gas sampling.

Drugs that induce methemoglobinemia either directly oxidize hemoglobin or are metabolically activated to an oxidizing species [7]. Phenazopyridine, used for relief of cystitis, can cause oxidative hemolysis [8]. Dapsone, used for leprosy, dermatitis herpetiformis, and prophylaxis for pneumocystis carinii, is metabolized to a hydroxylamine derivative [9]. It was the most common cause of methemoglobinemia in one recent series [10]. Primaquine and local anesthetics, such as topical or spray benzocaine (used prior to upper endoscopic procedures) and prilocaine, can cause methemoglobinemia [11–13]. Amyl nitrite and isobutyl nitrite have been implicated also [7]. Treatment includes cessation of the inducing agent, oxygen, and methylene blue.

## 5. Megaloblastic Anemia

Megaloblastic anemias are characterized by the presence of a hypercellular bone marrow with large, abnormal hematopoetic progenitor cells (megaloblasts). Leukopenia and thrombocytopenia also occur. Megaloblastic anemias can be congenital or acquired and most commonly are related to vitamin B_12_ (cobalamin) and folic acid deficiencies. While they are usually a result of malnutrition or defective absorption, they can also be drug-induced.

Drugs that act by interfering with DNA synthesis, such as antimetabolites and alkylating agents, some antinucleosides used against HIV and other viruses [14], can all induce megaloblastic anemia. Trimethoprim (in high, extended doses) and pyrimethamine, which bind with greater affinity to bacterial than human dihydrofolate reductase, have been associated with megaloblastic anemia, primarily among patients already at risk for folic acid deficiency. Antibiotics such as sulfasalazine and anticonvulsants such as phenytoin have been linked to folate-related changes which induce megaloblastic anemia, perhaps related to interference with absorption.

Decreased cobalamin levels have been reported with term use of histamine 2-receptor antagonists and proton pump inhibitors (e.g., omeprazole) [15, 16]. While protein bound B_12_ absorption may be impaired by these agents, clinically significant B_12_ deficiency seems rare despite widespread use.

## 6. Sideroblastic Anemia

Sideroblastic anemias (SAs) are characterized by ringed sideroblasts (erythroblasts containing iron-positive granules arranged around the nucleus) in the bone marrow. Sideroblastic anemias, which can be inherited or acquired, exhibit impaired heme biosynthesis in erythroid progenitors. Most sideroblastic anemias are acquired as clonal disorders of erythropoiesis. Additionally, ringed sideroblasts can be found in malnourished patients who abuse alcohol [17].

Drug-induced sideroblastic anemia has been associated with isoniazid [18]. The anemia is reversed by pyroxidine or by withdrawal of isoniazid. Chloramphenicol, rarely used at present, causes a reversible suppression of erythropoiesis and produces ringed sideroblasts [17]. Linezolide, penicillamine, and triethylene tetramine dihydrochloride (a chelating agent used to treat Wilson's disease) induce reversible SA [19–21]. Myelodysplasias and secondary acute leukemias induced by chemotherapy, discussed below, may initially manifest as sideroblastic anemia [22].

## 7. Aplastic Anemia

Aplastic anemia (AA), characterized by pancytopenia with a hypocellular bone marrow, can be inherited or acquired. Acquired aplastic anemia is most commonly idiopathic, but may be secondary to exposure to toxins, irradiation, viruses, and drugs. AA can develop as a direct response to exposure, but can also develop indirectly, through immune-mediated mechanisms. Historically, drug-induced AA has not been easily distinguished from idiopathic forms of the disease since with rare case reports causality is difficult to establish [23]. From an immunological perspective, the absence of antibodies in aplastic anemia suggests that drugs do not serve as simple haptens in the initiation of aplastic marrow failure.

Drugs implicated in inducing AA include antirheumatic drugs, antithyroid medications, antituberculous drugs, NSAIDs, and anticonvulsants. Specific drugs cited include chloramphenicol, butazone, sulfonamide, gold salts, penicillamine, amidopyrine, trimethoprim/sulfamethoxazole, methimazole, and felbamate [24–27].

Many drugs reported to cause aplastic anemia can also more commonly cause mild marrow suppression, suggesting that preliminary damage may occasionally (perhaps related to host metabolism) progress to more severe damage. The treatment and prognosis of drug-induced aplastic anemia seem similar to idiopathic cases [23].

## 8. Pure Red Cell Aplasia

Pure red cell aplasia (PRCA) is characterized by normocytic anemia, reticulocytopenia, and absence of mature marrow erythroid progenitors. PRCA is distinguished from aplastic anemia by relatively normal leukocyte and platelet counts. It can be congenital or acquired. Acquired PRCA can be idiopathic or secondary, either an acute, self-limiting disorder or a persistent, chronic refractory anemia. PRCA can arise in association with a thymoma, lymphoid cancer, parvovirus, rheumatoid arthritis, pregnancy, and drugs.

PRCA can be acquired through exposure to a number of drugs, including immunosuppressants (azathioprine, FK506, antithymocyte globulin), antibacterials (linezolide, isoniazid, rifampin, chloramphenicol), antivirals (interferon-alpha, lamivudine, zidovudine), fludarabine, anticonvulsants (diphenyldrantoin, carbamazepine, valporic acid), as well as chloroquine, allopurinol, ribavirin, and gold [28, 29].

Additionally, PRCA has been reported to develop after prolonged exposure to recombinant human erythropoietin (rHuEPO) specifically the brand Eprex, predominately used in Europe [30–33]. Withdrawal of rHuEPO followed by treatment with immunosuppressives (cyclosporine A) for several months rendered patients anti-EPO antibody negative and transfusion independent. PRCA seemed to occur predominantly with subcutaneous administration in renal failure patients. The frequency of this complication has reduced, seemingly as a result of changes in formulation and handling that may have decreased immunogenicity [33].

## 9. Immune Thrombocytopenia

In immune thrombocytopenia purpura (ITP), platelet destruction is caused as antibodies bind to platelets leading to their clearance by the reticuloendothelial system (RES), as well as by some degree of decreased production. ITP can be idiopathic, or related to viral infections, autoimmune disorders, lymphoproliferative disorders, or drugs [34, 35].

Classical causes of drug-induced thrombocytopenia are the quinine and quinine-like drugs [36]. The thrombocytopenia is typically sudden, severe, and may be accompanied by bleeding. The thrombocytopenia induced by these drugs is caused by antibody that is nonreactive in the absence of drug, but binds to epitopes on platelet membrane, glycoproteins IIb/IIIa or Ib/IX, when the sensitizing drug is present. Vancomycin can also be associated with marked thrombocytopenia and demonstrable drug-dependent antibodies in the serum [37]. Prolonged thrombocytopenia may occur in patients with renal insufficiency, likely due to delayed drug clearance. Other drugs associated with immune thrombocytopenia include include antimicrobials (sulfanomides, rifampin, linezolid), anti-inflammatory drugs, antineoplastics, antidepressants, benzodiazepines, anticonvulsants (carbamazepine, phenytoin, valproic acid) as well as cardiac and antihypertensive drugs [34, 35]. Although the ITP generally develops rapidly, it usually resolves upon cessation of treatment and is drug-specific.

Heparin is well known to be associated with thrombocytopenia, sometimes with arterial or venous thrombosis, which is generally a far greater threat than the risk of bleeding [38, 39]. Heparin-induced immune thrombocytopenia is caused by antibodies against complex of heparin and platelet factor 4 (PF4), which can lead to platelet activation and the initiation of thromboses. Although heparin can also induce a milder, nonimmune mediated thrombocytopenia, the immune version is potentially more severe. Heparin-induced thrombocytopenia follows exposure to both unfractionated and low molecular weight heparins, but is less common with the latter. There is usually a delay of 5–10 days for newly exposed patients, but thrombocytopenia can occur within hours in patients with a recent heparin exposure who still have PF4 antibodies, or within a few days for those with prior exposure who develop an anamnestic response. Occasionally venous gangrene, skin necrosis, and acute anaphylactic-type reactions to heparin can occur. In the appropriate clinical setting, the diagnosis is supported by evidence of antiheparin antibodies, which can be detected by a number of assays. These include the more sensitive serologic assays (e.g., by ELISA) and the functional, more specific assays such as measuring C-14 serotonin platelet release in the presence of heparin and serum. In addition to cessation of heparin use, treatment involves anticoagulation to reduce the risk of thrombosis, typically with argatroban, bivalrudin, or lepirudin initially, with transition to warfarin. Anticoagulation should be continued for several weeks even after the platelet count returns to normal due to the high risk of thrombosis during that time.

Abciximab (a chimeric Fab fragment) and eptifibatide and tirofiban (ligand mimetic inhibitors) are frequently used following coronary angioplasty to reduce thrombosis by impairing platelet function through the inhibition of GP IIb/IIIa-fibrinogen interaction. In addition to inducing the desired platelet dysfunction, however, they can induce a severe thrombocytopenia in a small percentage of patients, likely through a drug- dependent antibody-mediated mechanism [40]. This may begin within hours to days and typically resolves spontaneously in 2–5 days. It may occur on the initial or subsequent infusions. The majority of patients recover without complications though severe bleeding may occasionally occur. Platelet transfusions can be given if there is significant bleeding.

## 10. Thrombotic Microangiopathies

Thrombotic microangiopathies (TMAs) present with thrombocytopenia, microangiopathic hemolytic anemia, and symptoms of microvascular occlusion. They arise from excess platelet aggregation. TMA has often been associated with low levels of metalloprotease ADAMTS13, which induces cleavage of von Willebrand factor (vWF). Thrombotic thrombocytopenic purpura (TTP) and hemolytic-uremic syndrome (HUS) are the main thrombotic microangiopathies. TMA can be familial, idiopathic or acquired secondary to toxins, pregnancy, infections (e.g., HIV, certain Shigella and E. coli), and drugs.

Although drug-induced TMA is well-documented, its mechanism is not well-determined [41–44]. Immune-mediated or direct toxicity factors are most often proposed. In some patients with drug-induced TMA, autoantibodies to the ADAMTS13 protease are present, in some patients TMA incidence seems to be dose-dependent, and in many cases, there are no “hints” of mechanism at all.

One drug commonly implicated in inducing TMA is the immunosuppressant cyclosporine A (CyA). CyA-induced TMA is seen often in transplant patients (solid or liquid), but also in patients treated for rheumatoid arthritis and uveitis. The mechanism is believed to be a dose-related toxicity. TMA generally resolves once CyA treatment is reduced or discontinued.

Other drugs associated with TMA include chemotherapeutic agents mitomycin-C, gemcitabine, and cisplatin, as well as *α*-interferon and tacrolimus [41–43]. In patients with metastatic adenocarcinoma, it may be difficult to distinguish drug-induced TMA from anemia, thrombocytopenia, and microangiopathy related to carcinomatosis. Mitomycin-C is a known nephrotoxin, and there is evidence that its mechanism for inducing TMA is a dose-dependent, direct toxic effect on endothelium.

The thienopyridines, antiaggregating agents ticlopidine, and less commonly clopidogrel, have also been implicated in inducing TMA [44]. Ticlopidine-related TPA is more likely to occur after at least two weeks of therapy, to be associated with low ADAMTS13 levels with demonstrable auto-antibodies, and to benefit from plasma exchange therapy. Clopidogrel-induced TMA tends to occur within the first two weeks of treatment, is less likely to be associated with low ADAMTS13 levels and auto-antibodies, and is less likely to benefit from plasma exchange.

 Quinine may also induce TMA. The mechanism is immune-mediated. Patients with quinine-induced TMA have been found with antibodies against endothelial cells, lymphocytes, and granulocytes as well as quinine-dependent antibodies including IgG or IgM reactive with platelet glycoprotein Ib/IX or IIb/IIIa. TMA generally resolves with withdrawal of quinine along with plasma exchange [45].

## 11. Platelet Dysfunction

Disorders of platelet function may be detected in patients with prolonged bleeding times but normal platelet counts. While inducing platelet dysfunction to reduce the risk of thrombosis is often the desired purpose of some drugs (such as aspirin, clopidogrel, and anti-GP IIb/IIIa inhibitors), it may also be an undesired side effect [46].

Acetylation of cyclooxygenase 1 (COX 1) leads to impaired synthesis of the important platelet agonist thromboxane A_2_. Aspirin irreversibly acetylates COX 1, so that its effect persists even after the drug ceases to circulate. This is in contrast with nonselective nonsteroidal anti-inflammatory drugs, which reversibly acetylate COX 1. There is some evidence that aspirin has a dose-related effect on platelet aggregation [47].

 Fluoxetine and some tricyclic antidepressants, induce dysfunction by inhibiting serotonin uptake. Certain drugs can interfere with platelet adhesion or aggregation, including high dose pencillins and other *β*-lactam antibiotics, chemotherapy drugs such as mithramycin and daunorubicin, immunosuppresants, and phenothiazines [46].

## 12. Hypercoagulability

Hypercoagulability, with a propensity to both arterial and venous thrombosis, can be inherited or acquired. Hereditary thrombophilic conditions include factor V Leiden, prothrombin G20210A mutation, and deficiencies of proteins C, S or antithrombin III, among others. Acquired hypercoagulable states can be secondary to immobility, surgery, trauma, pregnancy, antiphospholipid syndrome, cancer, and drugs.

Selective COX-2 inhibitors, with less potential for bleeding and gastrointestinal toxicity than traditional COX inhibitors, became widely utilized as analgesics and anti-inflammatory agents and were also investigated for their potential effect of reducing the risk of polyps and colorectal cancer. However, celecoxib, rofecoxib, and valdecoxib were found to be associated with increased thrombotic cardiovascular events in several trials [48–50]. These results led to a voluntary withdrawal of rofecoxib from the worldwide market and focused intense scrutiny on pharmaceutical and FDA policies.

Erythropoietin has been associated with increased thrombotic risk. While erythropoietin markedly benefits the anemia of renal failure, targeting hemogloblin levels to high normal, it has been associated with higher cardiovascular morbidity and mortality than with lower target levels [51]. Questions have also been raised about the safety of erythropoietin in cancer patients. A meta-analysis did show increased thrombotic risk in treated patients, with some studies showing an increased risk of death [52]. Guidelines for more restricted and safer use of these agents in cancer patients are undergoing modification.

Hormonal therapies including oral contraceptives, hormone replacement therapy, and tamoxifen (a selective estrogen receptor modulator with some agonist activity) have all been associated with increased thrombotic risk. With oral contraceptives, risk of arterial and venous thrombosis may increase with age, genetic thrombophilias, smoking, and use of certain types of associated progestin [53]. The Women's Health Initiative study found that use of combined estrogen and progestin doubled the risk of venous thrombosis compared to placebo control [54]. For chemoprevention of breast cancer, raloxifene was found to have lower risk of thrombosis, as well as uterine cancer, than tamoxifen, and thus may be a safer drug in this setting [55]. Likewise, aromatase inhibitors such as anastrazole, letrozole, or exemestane used for treatment for early or advanced breast cancer demonstrate a lower thrombotic risk than tamoxifen [56].

 Adjuvant chemotherapy for breast cancer with CMF (cyclophosphamide, methotrexate, fluorouracil), an older regimen infrequently used presently, was associated with hypercoagulability [57–59]. Patients treated with CMF may have reduced levels of the inhibitors proteins C and S [57]. Thrombosis in patients receiving adjuvant chemotherapy may have become less common in part because of changes in types of chemotherapy used, a shorter duration of chemotherapy, the less frequent use of tamoxifen in postmenopausal patients, and the less frequent use of concomitant (as opposed to sequential) chemotherapy and tamoxifen [58].

Thrombotic complications have been associated with asparaginase, used to treat acute lymphoblastic leukemia [60–62]. Arterial and venous thromboses (including cerebral venous sinuses) can occur. L-asparaginase inhibits protein synthesis through the hydrolysis of the essential amino acid asparagine, causes reduced synthesis of antithrombin III, protein C, and protein S, thus leads to the increased thrombotic risk.

Thalidomide and lenalidomide, used for multiple myeloma, have been associated with increased thrombotic risk when used in combination with glucocorticoids [63]. Various prophylactic measures including warfarin, heparin, and aspirin have been recommended. The use of a less intensive once-weekly dexamethasone schedule decreases the thrombotic risk in comparison with the standard 4-day high dose schedule in combination with lenalidomide [63].

Drug-induced thrombosis from warfarin or heparin can be associated with skin necrosis. Warfarin-induced skin necrosis is often associated with preexisting thrombophilic conditions, such as protein C, S, or antithrombin III deficiency [64]. Therapy consists of discontinuation of warfarin, administration of vitamin K, and anticoagulation with heparin. However, heparin can also induce skin necrosis which may be part of the heparin-induced thrombocytopenia syndrome [65].

Bevacizumab, a monoclonal antibody against vascular endothelial growth factor, is used in metastatic colon, lung, and breast cancers has been associated with increased arterial thrombotic risk, particularly in elderly persons already predisposed to cardiovascular events [66].

## 13. Circulating Anticoagulants

Circulating anticoagulants inhibit clotting factors, causing excess hemorrhage. Autoantibodies such as acquired inhibitors to factor VIII may be idiopathic or secondary to hereditary hemophilia, the postpartum state, other autoimmune disorders, malignancies, or drugs. Patients present with bleeding as a syndrome of acquired hemophilia, with low F VIII: c levels and demonstrable F VIII inhibitors. Drugs implicated include antibiotics, psychotropics, fludarabine, and interferon [67]. Antibody activity resolves with cessation of the drug or with the use of immunosuppressive agents. An acquired inhibitor to factor XIII, which cross-links and stabilizes fibrin, has been associated with isoniazid [68].

Lupus anticoagulants and antiphospholipid antibodies may be induced by drugs such as chlorpromazine, hydralazine, phenytoin, quinine, and procainamide [69, 70]. In these cases, there is an association with hypercoagulability as opposed to bleeding.

## 14. Hypoprothrombinemia

Hypoprothombinemia, with prolongation of the PT/INR, is most commonly due to vitamin K deficiency or liver disease. Certain drugs have been linked with hypoprothrombinemia, such as broad spectrum antibiotics, usually in patients who are also malnourished. Reports have linked sulfonamides, ampicillin, chloramphenicol, tetracyclines, and cefoxitin to deficiency in vitamin K-dependent clotting factors [71]. Cephalosporins may be associated with hypoprothrombinemia, especially those with the N-methyl-thiotetrazole (NMTT) side chain (e.g., moxalactam, cefoperazone), although these are no longer in common use [72].

 For patients on warfarin, many drugs, especially antibiotics, are associated with increased hemorrhage. It is noted that there has been little systematic work on this subject, with the main sources being case reports [73]. Nonetheless, it is clear that many drugs interfere with coumadin through alteration of pharmacokinetics or dynamics (e.g., antibiotics, particularly quinolones, macrolides, and azoles), while others add to bleeding risk by their own mechanisms (e.g., aspirin, heparin, ticlopidine, and NSAIDs). Careful monitoring and dose adjustments are necessary when prescribing those medications to patients on warfarin.

## 15. Agranulocytosis/Neutropenia

Drug-induced neutropenia can occur in association with various analgesics, psychotropics, anticonvulsants, antithyroid drugs, antihistaminics, antirheumatics, GI drugs, antimicrobials, cardiovascular drugs, and, as expected, with chemotherapy drugs. [74–76]. Immune-mediated mechanisms are associated with some drugs such as penicillins which act as a haptens inducing antibody formation against neutrophils. Clozapine accelerates apoptosis of neutrophils, and propythiouracil causes complement mediated destruction of neutrophils. Drugs such as *β*-lactam antibiotics, carbamazepine, and valproate have a dose-dependent inhibition of granulopoiesis. Drugs with direct toxic effects on myeloid precursors include ticlopidine, bulsufan, methamizole, ethosuximide, and chlorpromazine.

Treatment of drug-induced neutropenia includes withdrawing the drug, antibiotic coverage when appropriate, and increasingly administration of recombinant human granulocyte colony-stimulating factor (rhG-CSF). The use of CSFs can reduce the duration of neutropenia, the frequency of infection, and possibly mortality, particularly in patients with profound neutropenia [76]. Mortality, although lower than in the past, remains about 5%.

 Rituximab, an anti-CD20 antibody, used in the treatment of B cell lymphoproliferative disorders and in benign autoimmune disorders, can induce neutropenia, typically of delayed-onset [77].

## 16. Neutrophilia

Neutrophilia can be related to myeloproliferative disorders but more commonly results from infection or inflammation. Drugs can also induce leukocytosis. Glucocorticoids cause neutrophilia by inducing the release of neutrophils from the bone marrow [78]. Although variable, glucocorticoids typically do not cause leukocytosis over 20 000/uL or a left shift. Such an elevation in WBC counts, or an increase in bands, might suggest the presence of infection [79]. Adrenergic-agonists and epinephrine produce neutrophilia by releasing neutrophils from the marginated pool [78]. Lithium causes mild neutrophilia and was used as treatment for neutropenia prior to the availability of CSFs [80].

Leukocytosis is commonly seen with G/GM-CSF, frequently given to reduce the severity and duration of neutropenia from chemotherapy. Sweet's Syndrome (acute febrile neutrophilic dermatosis), characterized by tender erythematous skin lesions, fever, and neutrophilia, can be induced by drugs such as trimethoprim-sulfamethoxazole, other antibiotics, and granulocyte colony stimulating factor, amongst others [81]. The syndrome can also be idiopathic or paraneoplastic.

## 17. Eosinophilia

Eosinophilia can result from both intrinsic hematologic disorders as well as secondary to a variety of systemic disorders and allergens, including drugs [82]. Drugs most commonly associated have included penicillins, sulfas, allopurinol, phenytoin, carbamazepine, and gold. Drug Rash with Eosinophilia and Systemic Symptoms (DRESS syndrome) describes the association of eosinophilia with rash, fever, and visceral involvement, such as pneumonitis, hepatitis, nephritis, adenopathy, or carditis [83]. DRESS has been associated most commonly with antibiotics, and anticonvulsants, but other agents have also been implicated.

## 18. Polycythemia

Polycythemia may be primary (polycythemia vera) or secondary to smoking, chronic hypoxia, certain tumors, or drugs. Drug-induced polycythemia can be seen with excess use of rHuEPO or anabolic steroids. Abuse of both types of agents by athletes may be associated with increased thrombotic risk [84]. Use of diuretics with volume contraction can contribute to pseudopolycythemia, where the hematocrit is elevated from hemoconcentration but true red cell mass is not increased.

## 19. Myelodysplasia and Acute Leukemia

Myelodysplastic syndromes (MDSs) and acute myeloid leukemia (AML) are clonal hematopoietic disorders associated with cytopenias, defective marrow maturation, and, ultimately, unregulated blast proliferation. Many cases of MDS evolve into AML, and although the two diseases are distinct, they share a continuous spectrum.

The majority of MDS and AML cases are idiopathic, but exposure to toxins and radiation can increase risk. Drugs can also induce MDS [85, 86]. Alkylating agents (such as nitrogen mustard, cyclophosphamide, melphalan, busuflan, chlorambucil) are the most frequently cited perpetrators. Risk has been related to total dose, duration, and specific type of alkylating agent. Procarbazine and nitrosoureas are also associated with myelodysplasia and leukemia. There can be a latent period of 2–8 years prior to development of *t*-MDS or AML. Leukemia induced by these agents is commonly preceded by a myelodysplastic syndrome. These leukemias are typically FAB M1 or M2 morphologically. Complex chromosomal abnormalities are typical, commonly with deletions of chromosome 5 and 7 and trisomy 8.

A distinct syndrome of secondary leukemia is related to topoisomerase II inhibitors, which includes the epipodophyllotoxins (etoposide and teniposide), anthracyclines (daunomycin, epirubicin, and doxorubicin.), and mitoxantrone. Leukemia with these agents has a shorter latency period than that associated with alkylating agents, often less than 2 years and usually presents without a prior MDS. Morphologically, acute myelomonocytic leukemias with a karyotypic abnormality involving 11q23 are commonly seen.

Treatment-related acute promyelocytic leukemia has also been described, most commonly in association with topo II inhibitors [87]. These also have a relatively short latency from treatment without a preceding preleukemic phase. Like de novo APL, *t*(15;17) with PML-RAR alpha rearrangements are common. Treatment outcomes are relatively favorable when treated like de novo APL, unlike the poor prognosis seen in other *t*-AML types.

 In patients receiving adjuvant chemotherapy for breast cancer, the risk of acute leukemia increases with age, with the intensity of therapy, and with the use of breast radiotherapy [88]. Many of these patients have received both alkylating agents and anthracyclines, both of which are leukemogenic. In addition, an increased risk of AML has been noted in breast cancer patients who received G-CSF along with adjuvant chemotherapy in some [89] but not all studies [90].

Stem cell transplantation, used in high risk, relapsed, and refractory hematologic malignancies, is associated with a risk of secondary MDS and leukemia. Whether the disease is induced by pretransplant therapy or the transplant itself remains uncertain.

Radioimmunotherapy, developed for non-Hodgkins lymphoma, may be associated with some risk of leukemogenesis. However, as with transplant patients, some of the risk may be related to stem cell damage from prior treatments or perhaps an increased risk related to the underlying disease itself. Small numbers of patients treated with radioimmunotherapy alone have shown a seemingly low risk for leukemia [93].

## 20. Conclusions

The wide spectrum of drug-induced hematologic syndromes is mediated by a variety of mechanisms, including immune effects, interactions with enzymatic pathways, and direct inhibition of hematopoiesis. The table summarizes the drug-induced hematological syndromes discussed above. Providing proof that a drug causes a particular hematologic syndrome is frequently impossible. Many patients simultaneously receive multiple drugs, making it difficult to be certain of causality. Rechallenge with a drug suspected of causing toxicity is usually not advisable. For some drugs, such as heparin, quinidine, and vancomyin, in vitro testing has been performed and mechanisms for cytopenias elucidated. However, such testing is not always possible given that for most there are no standardized, commercially available assays and that reactions may be related to metabolites as opposed to more easily tested parent compounds.

As medicine advances, older drugs become obsolete and are replaced by newer formulations. Many drugs formerly associated with hematologic toxicities (e.g., penicillin, alpha methyl-dopa, quinidine, gold, and chloramphenical) are no longer in common use. However, newer drugs are found to be associated with their own potential hematologic toxicities (e.g., clopidogel, linezolid, ribavirin, rituximab, and GPIIb/IIIa inhibitors). Furthermore, in addition to classic drug-induced cytopenias, we are increasingly seeing thrombosis as a common theme with a number of diverse agents (e.g., heparin, COX-2 inhibitors, bevacizumab, hormone replacement therapy, tamoxifen, erythropoietin, thalidomide and lenalidomide). Physicians from a wide variety of specialties need to understand the hematological consequences of drugs and be prepared for the occurrence and correction of these events in their patients.

## Figures and Tables

**Table 1:** Drug induced hematologic syndromes.

Syndrome	Examples of associated drugs	References
Immunohemolytic anemia	Pencillins, cephaloporins, alpha-methyl-DOPA, oxaliplatin, fludarabine, anti-Rh D antiglobulin	[1–4]
Nonimmune hemolytic anemia	Ribavirin, phenazopyridine, chloroquine,	[5, 6]
Methhemoglobinemia	Phenazopyridine, dapsone, benzocaine, prilocaine	[7–13]
Megaloblastic anemia	Rrimethoprim, pyrimethamine, diphenyhydantoin	[14–16]
Sideroblastic anemia	Isoniazid, chloramphenicol, linezolide	[17–22]
Aplastic anemia	Chloramphenical, gold, NSAIDs,	[23–27]
Pure red cell aplasia	Diphenylhydantoin, azathioprine, chlopropamide, isoniazid, erythropoietin	[28–33]
Immune thrombocytopenia	Quinine, quinidine, heparin, vancomycin, sulfas, pencillins, glycoprotein IIb-IIIa inhibitors	[34–40]
Thrombotic microangiopathy	Quinine, quinidine, clopidogrel, ticlopidine, cylosporine A, mitomycin-C, cisplatin	[41–45]
Platelet dysfunction	Pencillins, beta-lactam antibiotics, aspirin, NSAIDs	[46, 47]
Hypercoagulability	Estrogens, tamoxifen, asparaginase, heparin, bevacizumab, thalidomide/lenalidomide, COX-2 inhibitors, erythropoietin	[48–66]
Circulating anticoagulants	Isoniazid, hydralazine, procainamide	[67–70]
Hypoprothrombinemia	Cephalosporins, pencillins, sulfas	[71–73]
Neutropenia	Antithyroid drugs, procainamide, sulfas, captopril, phenothiazines, diphenylhydantoin, rituximab	[74–77]
Neutrophilia	Glucocorticoids, lithium, G- and GM-CSF	[78–81]
Eosinophilia	Pencillins, sulfas, allopurinol, diphenylhydantoin	[82, 83]
Polycythemia	Erythropoietin, anabolic steroids, diuretics	[84]
Acute leukemia/myelodyplasia	Alkylating agents, topoisomerase II inhibitors	[85–95]
